# Identification of Adjuvantic Activity of Amphotericin B in a Novel, Multiplexed, Poly-TLR/NLR High-Throughput Screen

**DOI:** 10.1371/journal.pone.0149848

**Published:** 2016-02-26

**Authors:** Alex C. D. Salyer, Giuseppe Caruso, Karishma K. Khetani, Lauren M. Fox, Subbalakshmi S. Malladi, Sunil A. David

**Affiliations:** 1 Department of Medicinal Chemistry, University of Kansas, Lawrence, Kansas, United States of America; 2 Department of Medicinal Chemistry, University of Minnesota, Minneapolis, Minnesota, United States of America; Centers for Disease Control and Prevention, UNITED STATES

## Abstract

Small-molecule agonists have been identified for TLR7, TLR8, TLR4 and TLR2 thus far, and chemotypes other than those of canonical ligands are yet to be explored for a number of innate immune receptors. The discovery of novel immunostimulatory molecules would enhance the repertoire of tools available for interrogating innate immune effector mechanisms, and provide additional venues for vaccine adjuvant development. A multiplexed, reporter gene-based high-throughput assay capable of detecting agonists of TLR2, TLR3, TLR4, TLR5, TLR7, TLR8, TLR9, NOD1 and NOD2 was utilized in screening 123,943 compounds, in which amphotericin B (AmpB) and nystatin were identified as prominent hits. The polyene antifungal agents act as TLR2- and TLR4-agonists. The TLR4-stimulatory activity of AmpB was similar to that of monophosphoryl lipid A, suggestive of TRIF-biased signaling. The adjuvantic activity of AmpB, at a dose of 100 micrograms, was comparable to several other candidate adjuvants in rabbit models of immunization. These results point to its potential applicability as a safe and effective adjuvant for human vaccines.

## Introduction

Vaccination against infectious diseases remains one of the most effective means of promoting human health [1, 2]. The World Health Assembly initiated a world-wide smallpox eradication program in 1966, leading to the eradication of smallpox [3, 4] from the planet, an *achievement [which] resulted not from possession of a magical new weapon such as a new vaccine*, *but from slight modifications in the use of a very old one*, *in fact*, *the oldest of all vaccines (P*. *Sartwell*, *prefatory remarks*, *Ref*. [3]*)*. There remain, however, a large number of devastating infectious diseases for which no effective vaccines currently exist, including diseases of enormous consequence to global health such as malaria, tuberculosis, and HIV/AIDS, as well as newly emerging pathogens. The major causes of mortality in pediatric populations in the developing world are attributable to lower respiratory infections, infectious diarrhea, malaria and measles [2], all of which are preventable illnesses. However, a significant impediment in the effective delivery of vaccines in the developing world is the requirement for most vaccines of multiple, spaced booster doses for successful immunization. Methods of safely enhancing immunogenicity of vaccines would be an important step toward realizing the bold [5, 6], but faltering [7] vision of the Children's Vaccine Initiative: an affordable, heat-stable, orally administered, multiple-antigen, single immunization to be given at birth.

In contrast to early vaccines which utilized inactivated whole organisms or attenuated live vaccines [8, 9], there is an increasing emphasis in contemporary vaccines on the use of subunit vaccines which have the distinct advantages of ease of production, quality control, and safety; however, such subunit antigens are largely soluble proteins and tend to be poorly immunogenic, necessitating the use of adjuvants to induce robust immune responses. The Food and Drug Administration (FDA) defines adjuvants as “agents added to, or used in conjunction with, vaccine antigens to augment or potentiate (and possibly target) the specific immune response to the antigen.” The addition of adjuvants can enhance vaccine effectiveness, especially for poorly immunogenic antigens, and may be of particular value in pediatric and geriatric populations, and in the immunocompromised [10–15]. Currently, the only vaccine adjuvants approved by the FDA are 'alum' (amorphous aluminum hydroxyphosphate sulfate) [16], introduced by Glenny in 1926 [17] and 3'-*O*-desacyl-4′-monophosphoryl lipid A, (MPLA), a Toll-like receptor 4 (TLR4) agonist derived from limited hydrolysis of Re-type lipopolysaccharide isolated from *Salmonella minnesota* Re595 [18–20]. The Strategic Plan for Research on Vaccine Adjuvants [21] developed by the National Institute of Allergy and Infectious Diseases (NIAID) provides guidance on vaccine adjuvant discovery and development, as well as clinical assessment of adjuvants, and intends to facilitate and promote the translation of early-phase adjuvant discovery to licensure of adjuvanted vaccines.

Macrophages and dendritic cells (DCs) function as immune sentinels against foreign antigens and pathogens. These cells recognize pathogen-associated molecular patterns (PAMPs) through pattern-recognition receptors (PRRs) and can induce innate immune responses which serve to marshal and amplify subsequent adaptive immune responses [22–27]. Vaccine adjuvants, including alum [28, 29] generally act via the engagement of PRRs. MPLA, as mentioned earlier [18–20], is an agonist of TLR4, one of ten trans-membrane, germline-encoded receptors in man [24, 30, 31]. TLRs, as well as Retinoic acid-Inducible Gene 1 (RIG-I)-like receptors [32] and Nucleotide-binding Oligomerization Domain (NOD)-like receptors (NLRs) [33, 34] constitute a growing number of PRRs [35–37]. The 10 functional TLRs in the human encode proteins with an extracellular domain having leucine-rich repeats (LRR) and a cytosolic domain called the Toll/IL-1 receptor (TIR) domain [24]. TLR1, -2, -4, -5, and -6 recognize extracellular stimuli, while TLR3, -7, -8 and -9 function within the endolysosomal compartment. The ligands for TLRs are highly conserved molecules such as lipopolysaccharides (LPS) (recognized by TLR4) lipopeptides (TLR2 in combination with TLR1 or TLR6), flagellin (TLR5), single stranded RNA (TLR7 and TLR8), double stranded RNA (TLR3), CpG motif-containing DNA (recognized by TLR9) [24].

Our focus on the discovery and development of safe and effective vaccine adjuvants has served as an impetus for a detailed exploration of structure-activity relationships in a variety of innate immune stimuli, including small molecule agonists of TLR2 [38–40], TLR7 [41–49], TLR8 [49–54], NOD1 [55], as well as C-C chemokine receptor type 1 (CCR1) [56]. Other than canonical ligands or derivatives thereof, defined small molecule agonists are yet to be discovered for a large number of PRRs such as TLR3 and TLR9, and it was of interest to us to embark on high-throughput screens with a view to identifying novel immunostimulatory molecular classes. Desiring a strategy that would permit the identification of immunostimulatory molecular classes acting on a very broad range of PRRs, we designed and evaluated a multiplexed, reporter gene-based high-throughput assay. Among the most prominent of 'hits' in screening 123,943 compounds were the polyene antifungal agents amphotericin B (AmpB) and nystatin. Deconvolution and dose-response profiles of the polyenes demonstrated TLR2- and TLR4-agonistic activity. Cytokine and chemokine induction profiles of AmpB closely resembled that of MPLA, suggesting a Toll–interleukin-1 receptor domain–containing adaptor inducing interferon-β (TRIF)-biased signaling. AmpB as an adjuvant was comparable to several other candidate adjuvants in rabbit models of immunization. These results point to its potential applicability as an adjuvant for human vaccines.

## Materials and Methods

### Compounds for HTS screens

**Fig 1:**
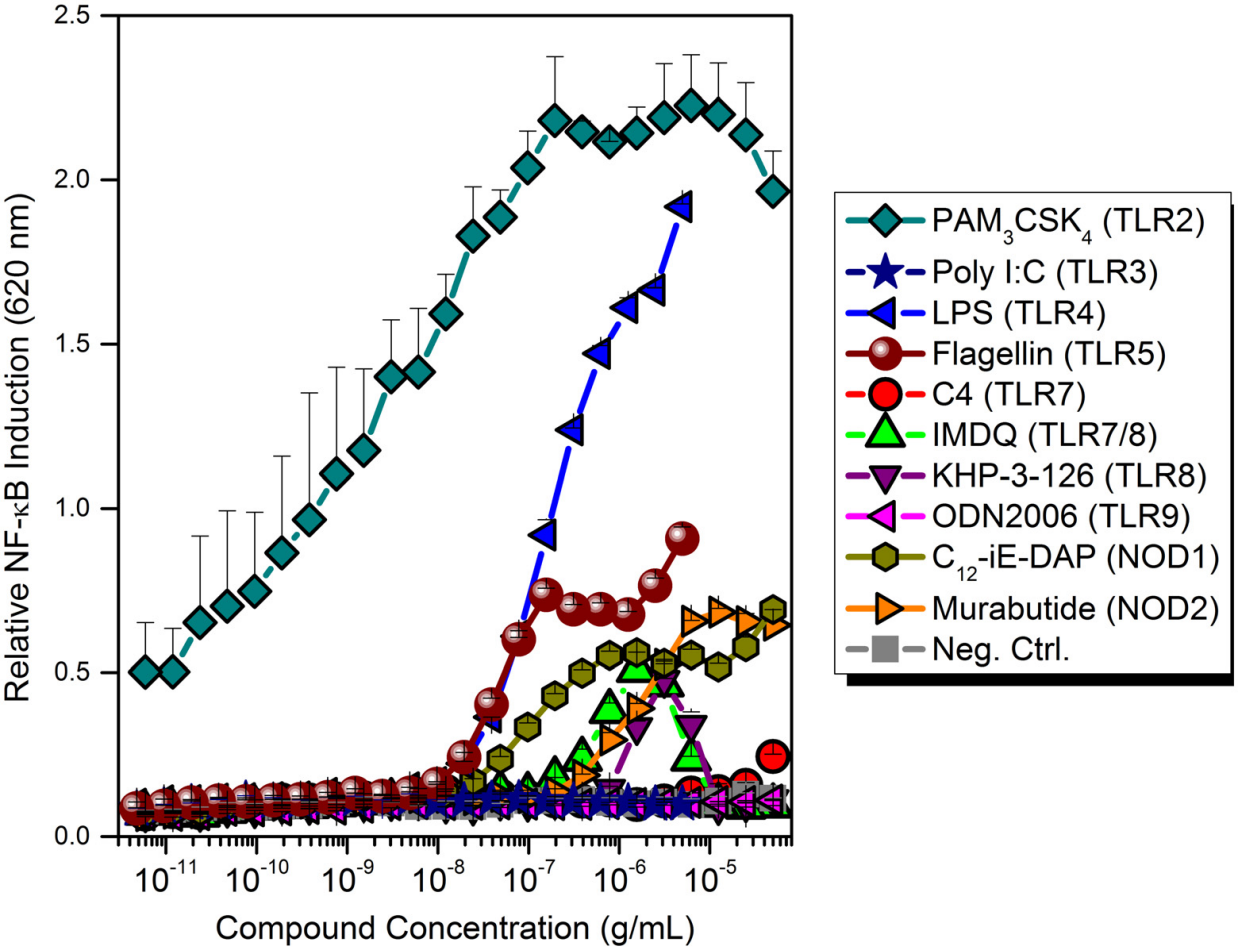
Responses of THP1-Blue^™^ NF-κB reporter cells to various TLR and NLR agonists. Dose-response profiles indicate strong responses to PAM_3_CSK_4_ (TLR2 agonist) and LPS (TLR4 agonist), and the absence of responsiveness to Poly I:C (TLR3 agonist), C4 (TLR7 agonist), and ODN2006 (TLR9 agonist). Attenuated responses were observed for flagellin (TLR5 agonist), KHP-3-126 (TLR8 agonist), C_12_-*i*E-DAP (NOD1 agonist) and Murabutide (NOD2 agonist).

Curated compound collections from the University of Kansas High Throughput Screening laboratory which include Life Chemicals (15,040), ChemBridge (43,736), ChemDiv (56,232), Selleck Bioactives (1649), TimTec (5000), and FDA Repurposed Library (2,286) were used. Compound transfers from source (80 nL of 10 mM stocks) to assay plates were performed using an Echo 550 acoustic liquid handler (Labcyte, Sunnyvale, CA). For most libraries, a target final concentration of 10 μM of compound (in a final volume of 80 μL for the multiplexed reporter gene-based assay described below) was achieved; the FDA Repurposed Library compounds were plated to obtain final concentrations of 2.5 μM. Assay plates were hermetically sealed and stored at -80°C until used. AmpB and nystatin were purchased from Sigma-Aldrich (St. Louis, MO). Synthetic MPLA, lipoteichoic acid (LTA) from *S*. *aureus*, PAM_2_CSK_4_, Poly(I:C), ultrapure LPS from *E*. *coli* K12, flagellin from *S*. *typhimurium*, ODN-2006 (Vaccigrade), C12-*i*E-DAP, and Murabutide were procured from InvivoGen (San Diego, CA). The structures of small molecule PRR agonists synthesized by us are shown in S1 Fig. Responses to a variety of TLR and NLR agonists were first examined using THP1-Blue^™^ NF-κB reporter cells (InvivoGen, San Diego, CA, Fig 1).

### Multiplexed human TLR-2/-3/-4/-5/-7/-8/-9/Null and NOD-1/NOD-2 reporter gene assays (NF-κB induction)

Human TLR-2/-3/-4/-5/-7/-8/-9/Null and NOD-1/NOD-2-specific reporter cells (InvivoGen, San Diego, CA; referred to hereafter as HEK2, HEK3 … HEK9 cells) were used for the multiplexed screen [42, 55, 56]. HEK293 cells stably co-transfected with the appropriate hTLR (or NOD) and secreted alkaline phosphatase (sAP) genes were maintained in HEK-Blue^™^ Selection medium (InvivoGen, San Diego, CA). Expression of sAP under control of NF-κB/AP-1 promoters is inducible by appropriate TLR/NOD agonists, and extracellular sAP in the supernatant is proportional to NF-κB induction.

**Fig 2:**
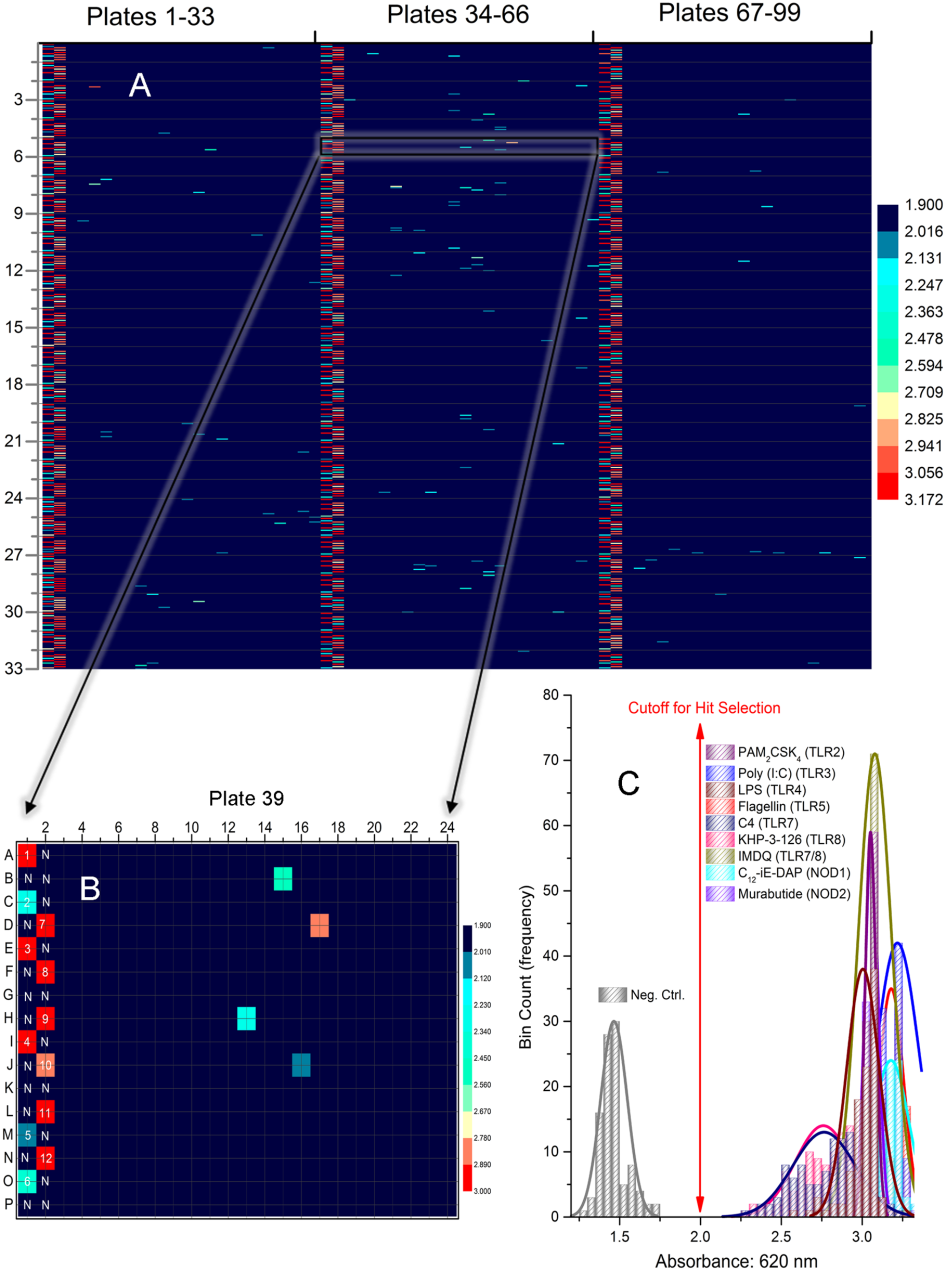
Pilot HTS Screen. A. Composite heat map of 99 384-well plates used for the pilot screen using the full complement of 10 multiplexed reporter cell lines. B. Heat map of Plate 39 from the pilot screen showing the organization of controls in the first two columns. Wells designated 'N' correspond to either medium alone or DMSO negative controls. Wells 1–11 correspond, respectively, to PAM_2_CSK_4_ (TLR2 agonist), Poly(I:C) (TLR3 agonist), LPS (TLR4 agonist), MPLA (TLR4 agonist), Flagellin (TLR5 agonist), C4 (pure TLR7 agonist), IMDQ (Dual TLR7/8 agonist), KHP-3-126 (pure TLR8 agonist), ODN-2006 (TLR9 agonist), C_12_-*i*E-DAP (NOD1 agonist), Murabutide (NOD2 agonist). All stimuli were used at a final concentration of 5 μg/mL (see S1 Fig for structures). Well 12 corresponds to a 'Master-Mix' which combined all the above-mentioned stimuli to yield an effective concentration of 0.45 μg/mL of the individual ligands. C. Distribution of baseline values and signals (a subset is shown for visual clarity). Means and standard deviations were computed for replicates for each of the stimuli from 99 plates from which Z' factors were calculated. Shown also are Gaussian fits of the histograms.

For the pilot screen, all ten individual reporter cells were used. Cells were harvested by trypsinization from T75 tissue culture flasks, washed once with pyrogen-free phosphate-buffered saline (PBS), and resuspended in HEK-Blue Detection Media at a density of 10^6^ cells/mL. Combining equal proportions of the ten different cell lines yielded a density of ~10^5^ cells/mL of each cell type. The first two columns of each assay plate were reserved for controls (see Fig 2B); alternate wells received DMSO or HEK-Blue Detection Media alone (unstimulated, negative controls), or individual, TLR/NOD-specific stimuli (see legend to Fig 2B). Structures of small-molecule TLR/NOD-specific compounds are shown in S1 Fig. 80 μl/well of the multiplexed cell mixture was added to 384-well, flat-bottomed, cell culture-treated assay plates, and incubated overnight in cell culture incubators. sAP was assayed spectrophotometrically using an alkaline phosphatase-specific chromogen (present in HEK-detection medium as supplied by InvivoGen) at 620 nm using a SpectraMax M4 multimode microplate reader (Molecular Devices, Sunnyvale, CA). Z' factors [57] were computed for each TLR/NOD-specific signal from 99 assay plates (Fig 2C).

The modified multiplexed assay used in the final screen included a subset comprising human TLR2, TLR4, TLR7, TLR8 and TLR9-specific reporter cells. The following individual stimuli were used to quantify signal-to-noise characteristics: PAM_2_CSK_4_ (TLR2), DBS-2-217c (TLR2) [39], lipoteichoic acid (LTA, TLR2), Poly(I:C) (TLR3), LPS (TLR4), flagellin (TLR5), C4 (pure TLR7) [42], EY-3-254B (pure TLR7) [48], IMDQ (TLR7/8) [43, 47], KHP-3-126 (pure TLR8) [53], MB-152 (pure TLR8) [54], ODN-2006 (TLR9), C_12_-*i*E-DAP (NOD1), and Murabutide (NOD2). Performance metrics for the controls used in the final screen are shown in S2 Fig.

All hits (n = 552), defined as signals ≤ 4 σ (in-plate standard deviations for test compounds above negative control means), were deconvoluted in full dose-response assays in human TLR-2/-3/-4/-5/-7/-8/-9 and NOD-1/NOD-2-specific reporter cells in liquid handler-assisted assay formats as described by us previously [42, 55, 56].

### Synthesis of pyridoxal phosphate adducts of AmpB and nystatin

AmpB (20.0 mg) was dissolved in 2 mL of dimethylformamide, to which was added a solution of 20 mg of pyridoxal phosphate in 2 mL of water (adjusted to pH 7.4 with Na_2_CO_3_). The mixture was lyophilized, and the resultant fully water-soluble adduct was characterized by liquid chromatography-mass spectrometry as described previously [58].

### Immunoassays for cytokines

Fresh human peripheral blood mononuclear cells (PBMCs) were isolated from human blood obtained by venipuncture (approved by the University of Minnesota institutional review board, Protocol ID: 1506-32702H). Written informed consent was obtained as per University guidelines. PBMCs were isolated using Vacutainer^®^ CPT^™^ Cell Preparation Tubes (Beckton-Dickinson, New Jersey, NJ). Aliquots of human PBMCs (10^5^ cells in 100 μL/well) were stimulated for 16 h with graded concentrations (two-fold dilutions starting at 25 μg/mL) of test compounds. Supernatants were isolated by centrifugation, and were assayed in triplicates (from individual donors) using analyte-specific multiplexed cytokine/chemokine bead array assays (Milliplex HCYTOMAG-60K, EMD Millipore, Billerica, MA) as reported by us previously [54]. The analytes examined include: sCD40L, VEGF, TNF-β, TNF-α, TGF-α, RANTES, PDGF-AB/BB, PDGF-AA, MIP-1β, MIP-1α, MDC (CCL22), MCP-3, MCP-1, IP-10, IL-17A, IL-15, IL-13, IL-12 (p70), IL-12 (p40), IL-10, IL-9, IL-8, IL-7, IL-6, IL-5, IL-4, IL-3, IL-2, IL-1ra, IL-1β, IL-1α, IFN-γ, IFN-α2, GRO, GM-CSF, G-CSF, Fractalkine, Flt-3 ligand, FGF-2, Eotaxin, EGF.

### Rabbit immunization and CRM197-specific immunoassays

All experiments were performed at Harlan Laboratories (Indianapolis, IN) in accordance with institutional guidelines (Protocol Number: 150416HBS73DAYSTD). The Harlan IACUC Committee approved this study. Small bore needles (24-gauge) were used to minimize distress to the animals during intramuscular administration. Following termination of the study, animals were first anesthetized using ketamine (0.5 mL of 100 mg/mL for average 6 lb. rabbit) and xylazine (0.5 mL of 20 mg/mL for average 6 lb. rabbit), and then euthanized by carbon dioxide inhalation. All antigen/adjuvant preparations were entirely aqueous; no liposomal or emulsifying agents were used. Cohorts of adult female New Zealand White rabbits (n = 4) were immunized intramuscularly in the flank region with (a) 10 μg of CRM197 [59] in 0.2 mL saline (unadjuvanted control), or (b) 10 μg of CRM197 in 0.2 mL saline plus 100 μg of either AmpB or TLR agonist. Pre-immune test-bleeds were first obtained via venipuncture of the marginal vein of the ear. Animals were immunized on Days 1, 15 and 28. A final test-bleed was performed via the marginal vein of the ear on Day 38. Sera were stored at −80°C until used. CRM197-specific ELISAs were performed in 384-well format using automated liquid handling methods as described by us elsewhere [47]. A Precision 2000 liquid handler (Bio-Tek, Winooski, VT) was used for all serial dilution and reagent addition steps, and a Bio-Tek ELx405 384-well plate washer was employed for plate washes. Nunc-ImmunoMaxiSorp (384-well) plates were coated with 80 μL of CRM197 (10 μg/mL) in 100 mM carbonate buffer, pH 9.0 overnight at 4°C. After 3 washes in 100 mM phosphate-buffered saline (PBS) pH 7.4, containing 0.1% Tween-20, the plates were blocked with 3% bovine serum albumin (in PBS, pH 7.4) for 1 h at rt. Serum samples (in quadruplicate) were serially diluted in a separate 384-well plate using the liquid handler; 40 μL of the serum dilutions were transferred using the liquid handler, and the plate incubated at 37°C for 1 h. The assay plate was washed three times, and 40 μl of 1:10,000 diluted appropriate anti-rabbit immunoglobulin (IgG, γ-chain) conjugated with horseradish peroxidase was added to all wells. Following an incubation step at 37°C for 1 h, and three washes, tetramethylbenzidine substrate was added at concentrations recommended by vendor (Sigma). The chromogenic reaction was terminated at 30 min by the addition of 2M H_2_SO_4_. Plates were then read at 450 nm using a SpectraMax M4 device (Molecular Devices, Sunnyvale, CA).

## Results and Discussion

Small-molecule agonists have been identified for TLR7 [41, 42, 47, 48, 60–67], TLR8 [49, 51–54, 68], TLR4 [69–72] and TLR2 [73, 74]. Chemotypes representing structural families other than those of canonical ligands are yet to be explored for a number of TLRs such as TLR3, TLR5 and TLR9, as well as other PRRs. The discovery of novel immunostimulatory molecules of defined receptor specificities would enhance the repertoire of tools available for interrogating innate immune effector mechanisms, and provide additional venues for vaccine adjuvant development. Our primary goal was, therefore, to identify novel PRR agonists. We envisioned that an efficient strategy would be to design and implement an assay that would permit the identification of immunostimulatory molecular classes acting on a very broad range of PRRs and, having cast a wide net, as it were, to then deconvolute signals and assign receptor specificities to such molecules.

We tested this premise by first examining the responses of a human monocyte-derived THP-1 reporter cell line; similar constructs have been successfully utilized to identify agonists of TLR4 [69, 71]. These cells responded robustly to TLR2 and TLR4, and feebly to TLR5, TLR8, NOD1 and NOD2 stimuli; agonists of TLR3, TLR7 and TLR9, however, failed to elicit any response (Fig 1). We therefore set out to develop a ‘Universal Reporter’ cell line by constructing hybridomas derived from the fusion of CD14^+^ primary human monocytes with human embryonic kidney cells (HEK) expressing only the NF-κB-inducible secreted embryonic alkaline phosphatase (sAP) reporter gene (HEK-Blue^™^ Null cells) [56]. Although we were able to isolate, expand and characterize the ploidy of the heterokaryons, these cells were unstable, rapidly regressing back to an embryonic, CD14^-^ phenotype (data not shown).

We reasoned that multiplexing (combining) individual reporter cell lines (HEK2, HEK3, HEK4, HEK5, HEK7, HEK8, HEK9, NOD1, NOD2 and HEK-Null) would also achieve our objective of simultaneously detecting signals from a wide range of PRRs. Utilizing this multiplexing approach, we conducted a pilot high-throughput screen of 34,848 compounds (Fig 2A). Z’ factors [57] were optimized by varying several parameters (relative proportion of each reporter cell line, total cell density per well, incubation period, liquid handling protocols and plating methods). Responses in this assay were first characterized using both a ‘Master Mix’ (mixture of individual ligands), as well as individual stimuli (a metric for deconvolution) in each of the ninety-nine test plates (Fig 2B). Although excellent Z' values were observed to individual stimuli (Fig 2C), the relatively high baselines in unstimulated control wells (median: 1.5 AU, Fig 2C), necessitated a cutoff value of 2.0 AU, which could potentially limit the dynamic range of the assay and possibly compromise the detection of weak signals.

**Fig 3:**
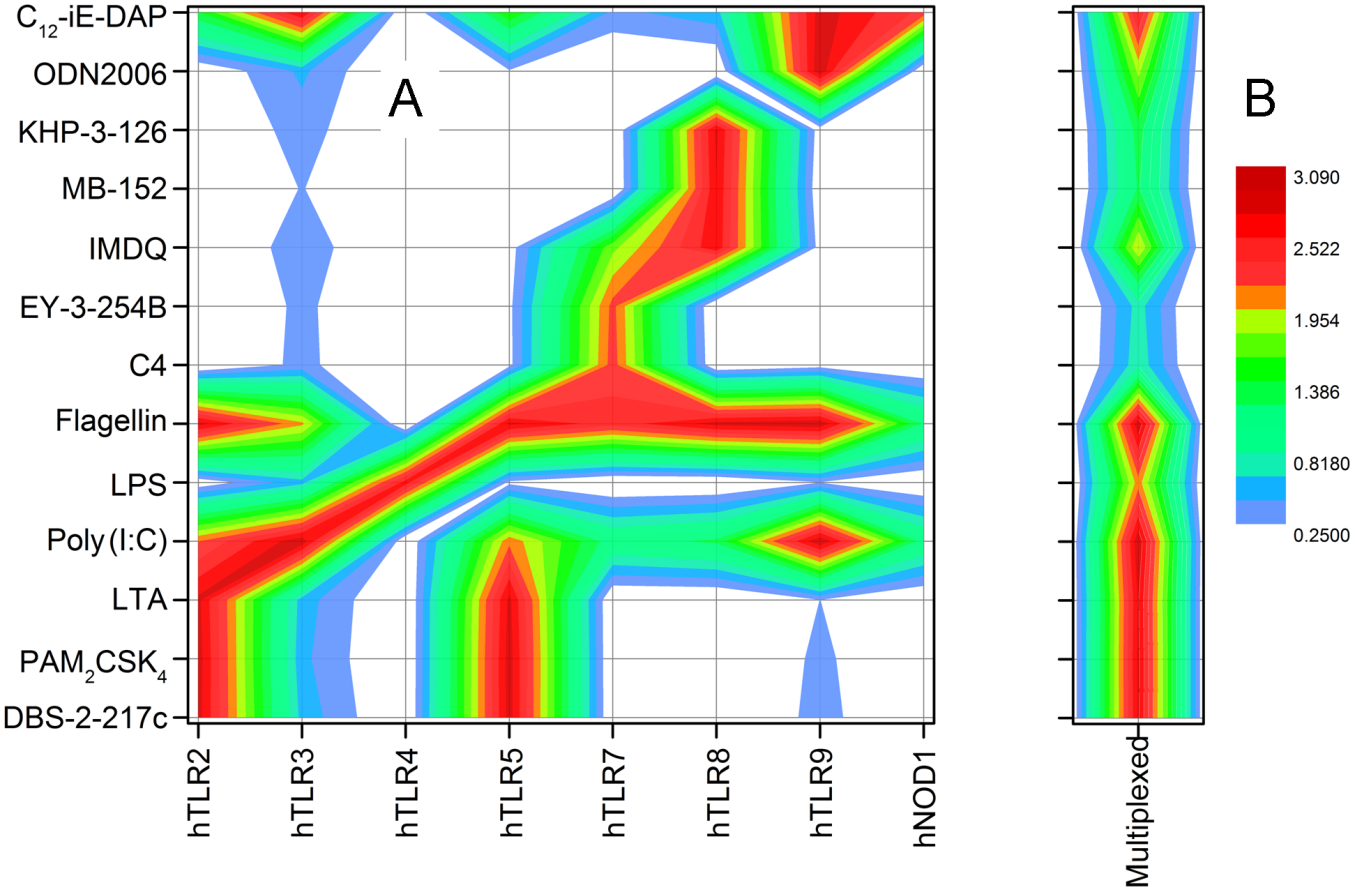
Responses of individual reporter cell lines to innate immune stimuli. A. Heat map of responses of individual reporter cell lines to a variety of TLR/NLR stimuli (see S1 Fig for structures of compounds), showing redundancy in PRR engagement. B. Multiplexing TLR2, TLR4, TLR7, TLR8, and TLR9 cells is sufficient and necessary for robust detection of signals.

We examined the possibility of improving signal-to-noise (S/N) ratios and dynamic range of the assay by eliminating possible redundancy in signaling in the individual reporter cell lines. We tested the responses of each individual reporter cell line to well-characterized innate immune stimuli. We observed considerable redundancy and degeneracy within these reporter cells (Fig 3A). Virtually all of the reporter cell lines responded to Poly(I:C), a TLR3 ligand which also signals via the RIG-I-like receptors, RIG-I, Melanoma Differentiation-Associated protein 5 (MDA5), or the RIG-I-like RNA helicase LGP2 [32, 75, 76]. HEK2 cells responded, as expected, to canonical TLR2 ligands (PAM_2_CSK_4_ [77], LTA [78], and the monoacyl human TLR2-specific lipopeptide, DBS-2-217c [39]), but also to flagellin and C_12_-*i*E-DAP [55], TLR5 and NOD1 agonists, respectively; HEK5 cells responded, in reciprocal fashion, to flagellin and the TLR2 agonists, as well as C_12_-*i*E-DAP. Flagellin also activates, apparently ectopically, HEK3, HEK4, HEK7, HEK8, HEK9 and NOD1 cells, presumably by alternate sensing via Naip5 [79, 80]. HEK7 cells responded to pure TLR7 agonists C4 [42], EY-3-254B [48] and to the dual TLR7/8 agonist IMDQ [43, 47] (in addition to, as mentioned earlier, poly(I:C) and flagellin), as well as to C_12_-*i*E-DAP. HEK8 cells showed responses to TLR8 agonists KHP-3-126 [53], MB-152 [54], and to the dual TLR7/8 agonist IMDQ; poly(I:C), flagellin and C_12_-*i*E-DAP also elicited NF-κB induction in these cells. An analysis of the redundancies indicated that a subset comprising HEK2, HEK4, HEK7, HEK8 and HEK9, when multiplexed, responded to all of the innate immune stimuli (Fig 3B), while showing a markedly reduced baseline absorbance of 0.25 AU.

**Fig 4:**
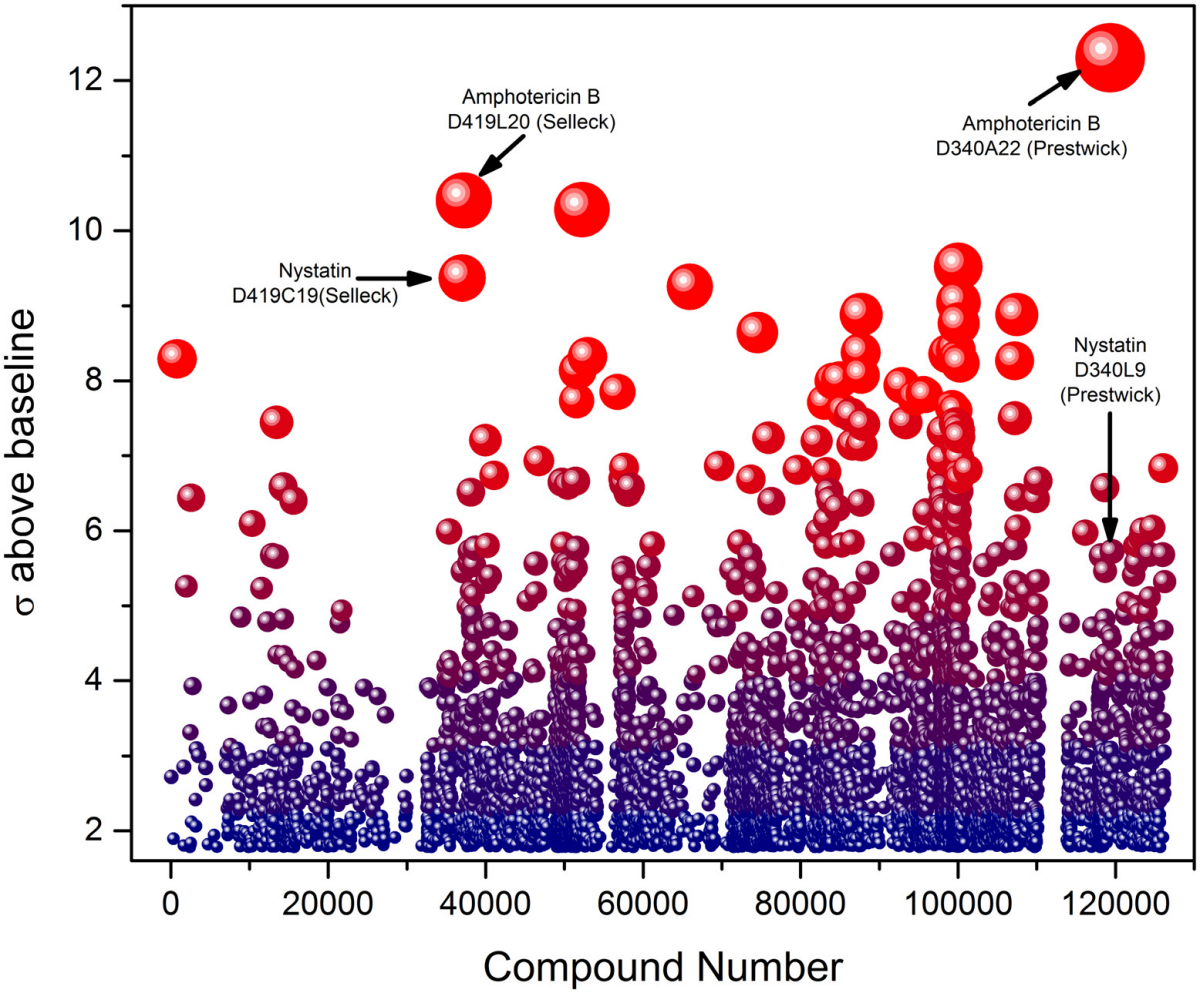
HTS data on 123,943 compounds showing prominent signals originating from AmpB and nystatin.

This multiplexed platform was implemented in screening to screen 123,943 compounds and, as in the pilot screen, the inclusion of individual controls in each of the 354 assay plates (Fig 2B) allowed the examination of S/N ratios and Z' factors for individual stimuli, which ranged from 0.74 (HEK7) to > 0.85 for poly(I:C) and flagellin (S2 Fig). A cutoff value of 4σ above in-plate absorbance mean yielded 552 provisional hits (Fig 4). Among the most prominent of signals were those originating from the polyene antifungal agents AmpB and nystatin; both these compounds were identified as prominent hits in the Selleck as well as the Prestwick libraries (Fig 4).

**Fig 5:**
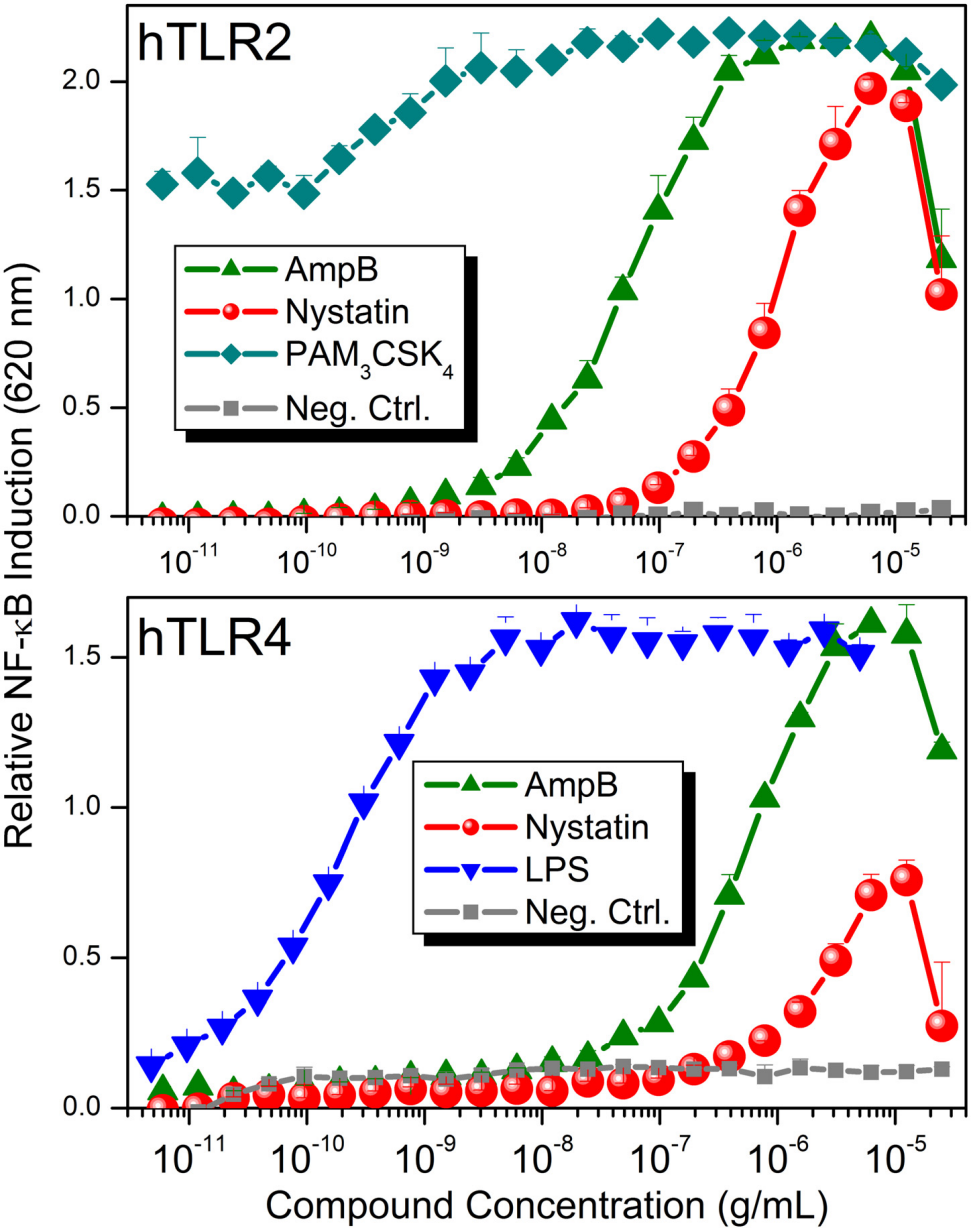
Deconvolution and dose-response of AmpB and nystatin showing human TLR2 and TLR4-agonistic activities.

Simultaneous deconvolution and dose-response profiles in individual reporter cell lines (HEK2, HEK3, HEK4, HEK5, HEK7, HEK8, HEK9, NOD1 and HEK-Null) were performed for all provisional hits. AmpB and nystatin showed dose-dependent NF-κB induction in human TLR2- and TLR4-specific reporter cell lines (Fig 5), consistent with previous reports demonstrating TLR2 and TLR4 activation by these antifungal agents [81–83].

AmpB has remained the frontline chemotherapeutic agent for serious systemic fungal infections for more than a half-century [84, 85]. AmpB is, as the name suggests, amphoteric which, compounded by its pronounced amphipathic nature (due to the asymmetric distribution of polar hydroxyl groups on face of the molecule and a markedly hydrophobic, conjugated polyene on the other), has a marked propensity to self-associate with a critical aggregation concentration of ~ 0.2 μg/mL. Consequently, the drug is very sparingly soluble in water (< 1 μg/mL) [58]. AmpB was more potent than nystatin (Fig 5), is an FDA-approved drug for parenteral use (which nystatin is not), and we had previously reported a practical and convenient method for obtaining highly water-soluble (>100 mg/mL) formulations of AmpB using pyridoxal phosphate (vitamin B_6_) as a complexing agent (structure shown in S1 Fig) [86]. For these reasons, we chose to evaluate the adjuvantic properties of AmpB.

**Fig 6:**
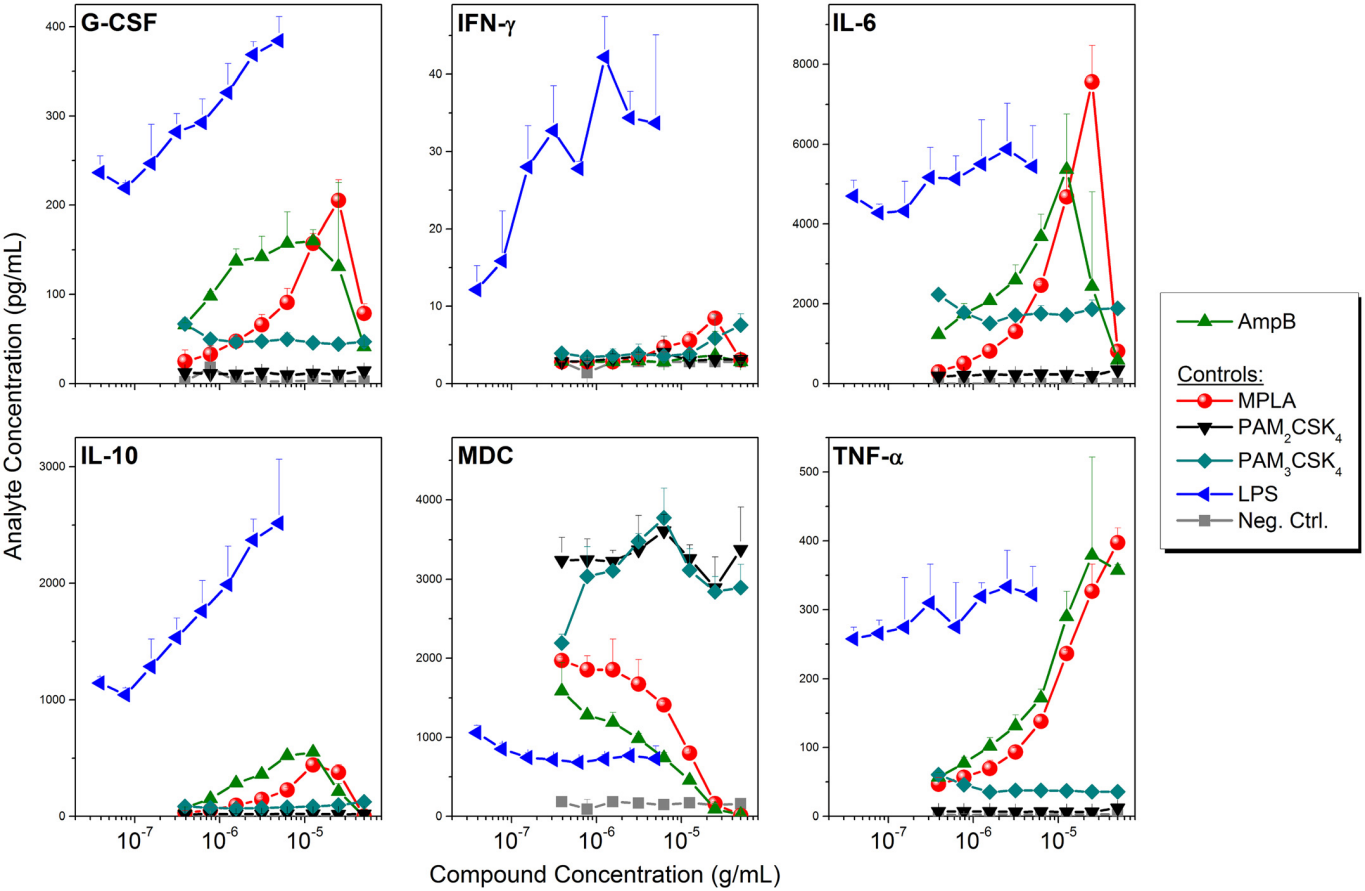
Cytokine and chemokine induction of AmpB in human PBMCs. Aliquots of human PBMCs (10^5^ cells in 100 μL/well) were stimulated for 16 h with graded concentrations (two-fold dilutions starting at 25 μg/mL) of test compounds. Supernatants were isolated by centrifugation, and were assayed in triplicates (from individual donors) using analyte-specific multiplexed cytokine/chemokine bead array assays. The cytokine profile of AmpB closely resembles that of MPLA, suggestive of TRIF-biased signaling (see S3 Fig for additional cytokine and chemokine responses).

Given the dose-dependent TLR2- and TLR4-agonistic properties [81–83] of AmpB (Fig 5), we compared cytokine and chemokine induction by AmpB to the TLR2-specific diacyl and triacyl lipopeptides PAM_2_CSK_4_ and PAM_3_CSK_4_ (which signal via TLR2/6 and TLR2/1 heterodimerization, respectively [87, 88]), MPLA, a TLR4 agonist which is a component of the FDA-approved AS04 adjuvant [18–20], as well as LPS, a highly proinflammatory TLR4 agonist, in human PBMCs. A high degree of congruence in the pattern of cytokine and chemokine induction between AmpB and MPLA was observed (Fig 6), suggesting a TRIF-biased signaling for AmpB, as has been observed for MPLA [89–91]. In particular, both AmpB and MPLA, relative to LPS, induce significant levels of Myd88-dependent TNF-α and IFN-γ secretion [89] only at high concentrations, whereas both AmpB and MPLA strongly induce MDC (Fig 6) and RANTES (S3 Fig) responses at low concentrations, consistent with TRIF-dominant signaling of AmpB [89–91]. A possible clinical correlate of a dominant TRIF-biased TLR4 activation by AmpB, which is associated with relatively low levels of proinflammatory mediator in circulation, is the occurrence of infusion-related febrile reactions [84, 92] at therapeutic doses of up to 1 mg/kg per day; in contrast, febrile reactions and hemodynamic derangements in humans occur at much lower doses of LPS infusion (4 ng/kg) [93–95].

**Fig 7:**
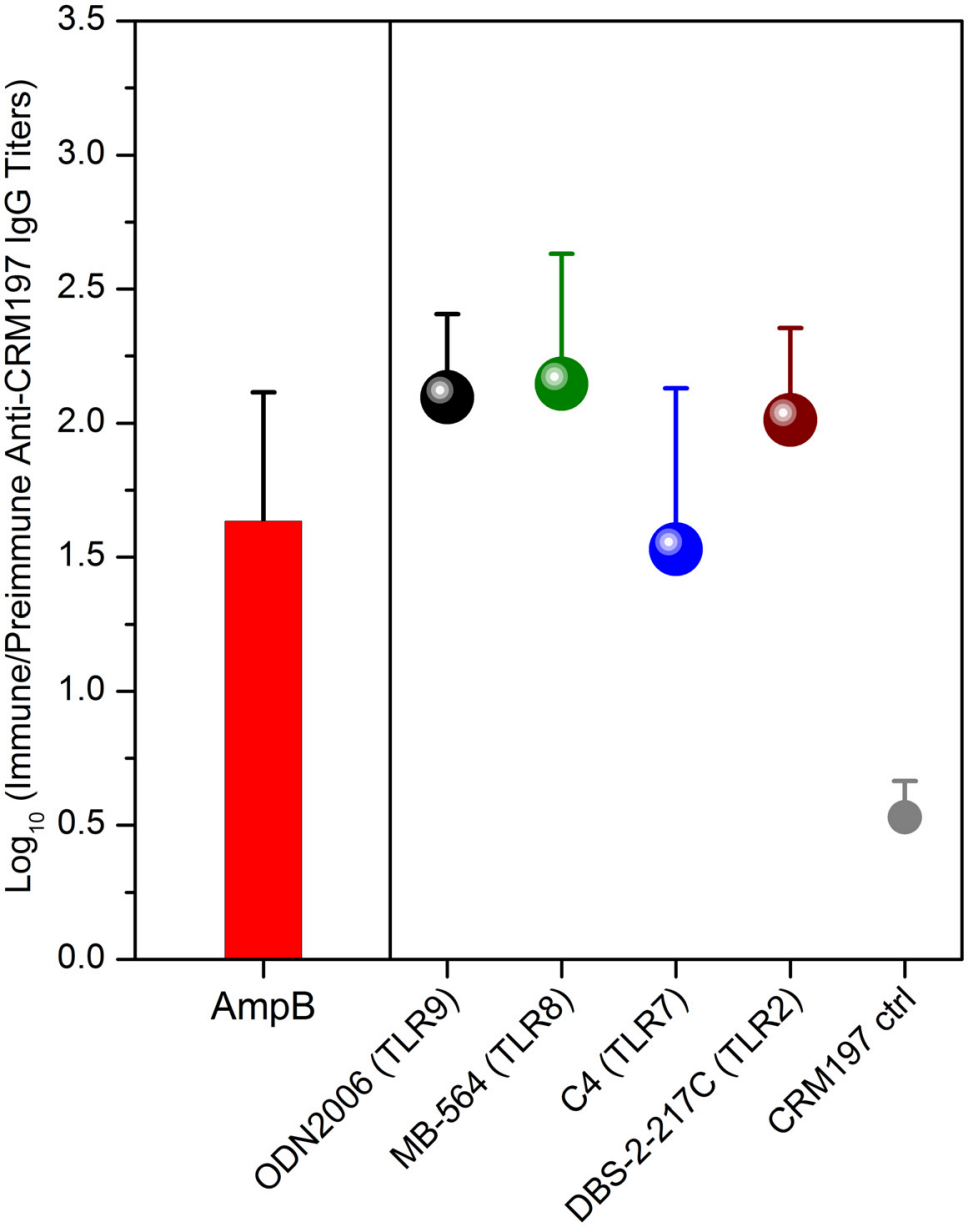
Adjuvanticity of AmpB in a standardized rabbit model of immunization. Cohorts of adult female New Zealand white rabbits (n = 4) were immunized intramuscularly in the flank region with 10 μg of CRM197 in 0.2 mL of saline (unadjuvanted control) or 10 μg of CRM197 in 0.2 mL of saline plus 100 μg of AmpB-pyridoxal phosphate adduct without any other excipients. Other TLR-active candidate adjuvants were used as comparators. Preimmune test-bleeds were obtained on day 0, and animals were immunized on days 1, 15, and 28. A final bleed was obtained on day 38. CRM197-specific ELISAs were performed using automated liquid handling methods and are depicted as log_10_ (immune/preimmune) titers.

The *in vitro* signatures of a TRIF-biased cytokine induction profile that portend low, if any, local or systemic reactogenicity, coupled with the fact that parenteral AmpB has been in the clinic for several decades prompted the evaluation of its adjuvanticity in highly standardized rabbit models [54, 68] that we have been using to benchmark candidate vaccine adjuvants using CRM197 (10 μg/dose) as antigen [59]. CRM197 is a non-toxic mutant of diphtheria toxin, containing a single amino acid substitution (G52E), and is used as a carrier protein in conjugate vaccines for polysaccharide antigens, which are frequently T cell-dependent antigens, requiring T cell help for humoral responses [59]. The adjuvantic effects of the highly water-soluble and stable AmpB-pyridoxal phosphate complex (100 μg/dose) was compared to that of ODN 2006 (TLR9), MB-564 (TLR8), C4 (TLR7) and DBS-2-217c (TLR2), all of which are also fully water-soluble and were also used at 100 μg/dose. AmpB as an adjuvant elicited comparable anti-CRM197 IgG titers relative to the other candidate adjuvants (Fig 7) in rabbits with no detectable local inflammation. As mentioned earlier, AmpB has had a long track-record in the clinic, with a well documented adverse effect profile; the very low doses of AmpB required for adjuvanting vaccines emphasizes its potential use as an adjuvant for human vaccines. Furthermore, the development of a novel multiplexed innate immune detection platform described herein has led to the identification of several immunostimulatory chemotypes, structure-activity relationship studies of which are currently in progress, and will be reported elsewhere.

## Supporting Information

S1 Fig Structures of small-molecule TLR agonists used as individual controls; also shown is the structure of the water-soluble adduct of amphotericin B with pyridoxal phosphate. (DOCX)

S2 Fig Distribution of negative and individual positive controls obtained in the modified multiplexed HTS screen. (DOCX)

S3 Fig Comparison of cytokine and chemokine induction profiles by AmpB, MPLA,LPS, PAM_2_CSK_4_, and PAM_3_CSK_4_. The following analytes did not show significant responses: Eotaxin, TGF-β, GRO, IL-12p70, PDGF-AA, IL-13, IL-15, sCD40L, IL-17α, IL-9, IL-2, IL-3, IL-5, IL-7, TNF-α.
(DOCX)
