# Supplementary material for: Identification of Adjuvantic Activity of Amphotericin B in a Novel, Multiplexed, Poly-TLR/NLR High-Throughput Screen
Source: PLoS One. 2016 Feb 26;11(2):e0149848. doi: 10.1371/journal.pone.0149848 (PMC4769227; doi:10.1371/journal.pone.0149848)
Supplement: S1 Fig — (DOCX) [file pone.0149848.s001.docx]

**S1 Fig.** **Structures of small-molecule TLR agonists used as individual controls; also shown is the structure of the water-soluble adduct of amphotericin B with pyridoxal phosphate**.
