# Supplementary material for: Identification of Adjuvantic Activity of Amphotericin B in a Novel, Multiplexed, Poly-TLR/NLR High-Throughput Screen
Source: PLoS One. 2016 Feb 26;11(2):e0149848. doi: 10.1371/journal.pone.0149848 (PMC4769227; doi:10.1371/journal.pone.0149848)
Supplement: S2 Fig — (DOCX) [file pone.0149848.s002.docx]

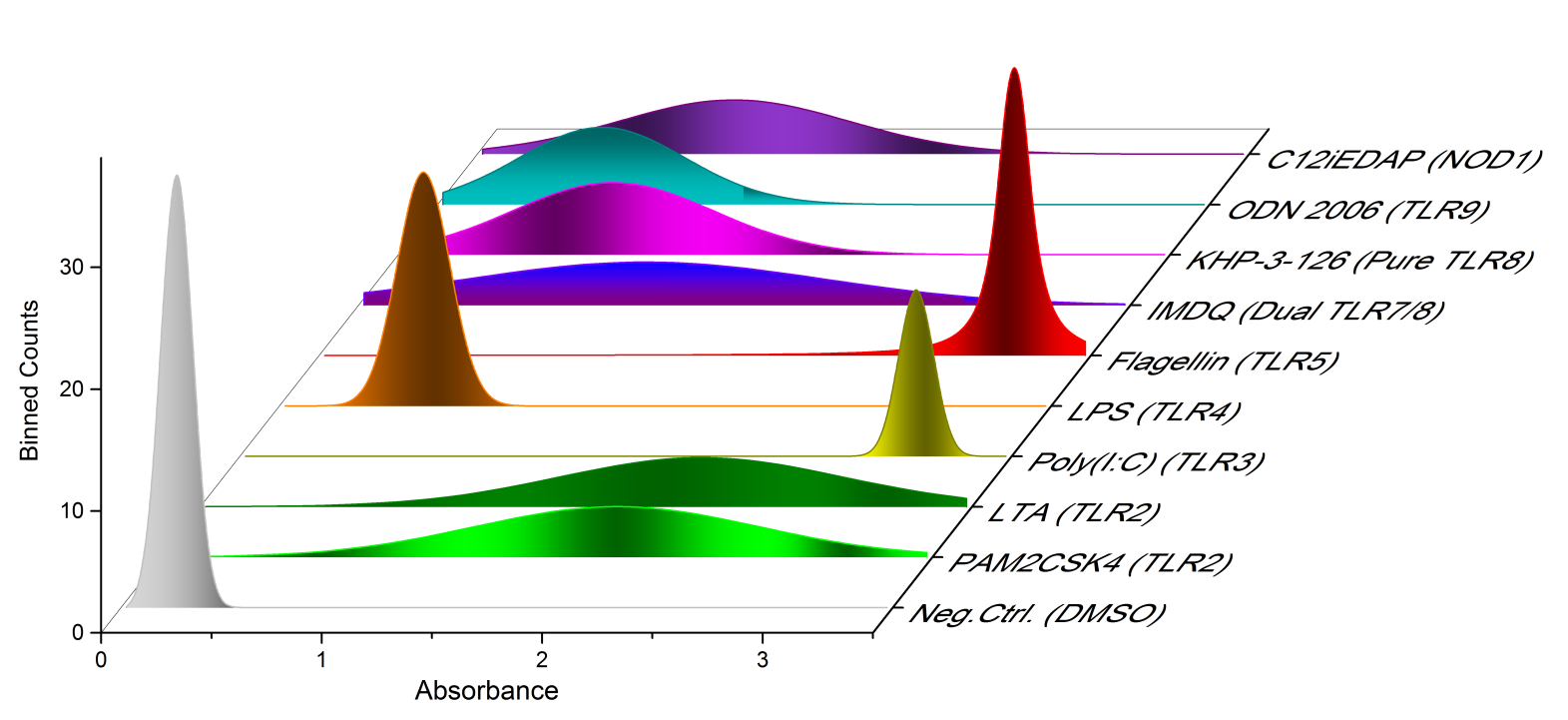


**S2 Fig. Distribution of negative and individual positive controls obtained in the modified multiplexed HTS screen.**
