# Supplementary material for: Identification of Adjuvantic Activity of Amphotericin B in a Novel, Multiplexed, Poly-TLR/NLR High-Throughput Screen
Source: PLoS One. 2016 Feb 26;11(2):e0149848. doi: 10.1371/journal.pone.0149848 (PMC4769227; doi:10.1371/journal.pone.0149848)
Supplement: S3 Fig — The following analytes did not show significant responses: Eotaxin, TGF-β, GRO, IL-12p70, PDGF-AA, IL-13, IL-15, sCD40L, IL-17α, IL-9, IL-2, IL-3, IL-5, IL-7, TNF-α. (DOCX) [file pone.0149848.s003.docx]

**S3 Fig. Comparison of cytokine and chemokine induction profiles by AmpB, MPLA,LPS, PAM_2_CSK_4_, and PAM_3_CSK_4_**. The following analytes did not show significant responses: Eotaxin, TGF-α, GRO, IL-12p70, PDGF-AA, IL-13, IL-15, sCD40L, IL-17α, IL-9, IL-2, IL-3, IL-5, IL-7, TNF-β.
